# Characterization and Thermal Stability of Acetylated Slicewood Production by Alkali-Catalyzed Esterification

**DOI:** 10.3390/ma10040393

**Published:** 2017-04-07

**Authors:** Ke-Chang Hung, Chen-Ning Yang, Teng-Chun Yang, Tung-Lin Wu, Yong-Long Chen, Jyh-Horng Wu

**Affiliations:** Department of Forestry, National Chung Hsing University, Taichung 402, Taiwan; d9833004@mail.nchu.edu.tw (K.-C.H.); abcyjn96@hotmail.com (C.-N.Y.); tcyang.04@nchu.edu.tw (T.-C.Y.); tonywuwu22@gmail.com (T.-L.W.); d9733202@mail.nchu.edu.tw (Y.-L.C.)

**Keywords:** acetylation, slicewood, vinyl acetate, thermal decomposition kinetic, apparent activation energy

## Abstract

This study was compared and characterized two different alkali (potassium carbonate (PC) and potassium acetate (PA))-catalyzed acetylations of slicewood with vinyl acetate (VA) by a vapor phase reaction. The results revealed that the esterification reaction between VA and the hydroxyl groups of slicewood could be improved by using PC or PA as a catalyst. Additionally, a significant weight percent gain was obtained after VA acetylation with 5% of catalyst. Furthermore, the reactivity of the cellulose hydroxyl groups for VA acetylation was more pronounced at the C2 reactive site compared to acetylation with acetic anhydride. On the other hand, the apparent activation energy of thermal decomposition between 10% and 70% conversion is 174–183, 194–200, and 183–186 kJ/mol for unmodified slicewood and VA-acetylated slicewood with PC and PA, respectively. Accordingly, the thermal stability of the slicewood could be effectively enhanced by VA acetylation, especially for using the PC as a catalyst.

## 1. Introduction

In recent years, slicewood and veneer have become a more important part of the wood market because the gradual disappearance of global forest resources has let the supply of large trees to diminish [1]. However, slicewood or veneers are often sourced from fast-grown plantation forests, with properties and durability inferior to those sourced from natural-growth forests. Several physical and chemical methods, such as acetylation, heat treatment, and sol-gel treatment, have been used to improve the properties and durability of wood [2,3,4,5]. Among these known approaches, acetylation with acetic anhydride (AA) is one of the best chemical modifications, with corresponding products having already been commercialized in the USA, Europe, and Japan [6,7,8,9].

The conventional acetylation process is not only time-consuming, but also requires large quantities of the modifying agent. According to Yang et al. [10], wood acetylation with AA using a vapor phase reaction could reduce the reagent consumption. However, using AA as a reactant has a main drawback of generating acetic acid as a by-product during the reaction, which results in undesirable odors and is difficult to remove from wood after acetylation due to its high boiling point (ca. 78 °C) [3,6,7,11,12]. More recently, Jebrane and Sèbe showed that wood was successfully acetylated by an alternative modifying agent using a vinyl acetate (VA) transesterification [12]. The main advantages of this approach are a non-acidic by-product and low boiling point (21 °C). However, very few studies have addressed wood acetylation with VA using a vapor phase reaction. Furthermore, there is very little information about the influence of catalyst types on the reactivity of VA and the physico-mechanical and viscoelastic properties of VA-acetylated wood. 

On the other hand, the thermal stability of the acetylated wood is mostly estimated by thermogravimetric analyses (TGA) [5,13,14,15]. More recently, the “model-free” iso-conversion methods have been widely employed to determine the activation energy (*E*_a_) of materials. These approaches allow the observation of the *E*_a_ dependence without assuming the reaction function and reaction order with the same conversion of the thermogravimetric (TG) curves estimated at different heating rates [16,17]. These models provide a quantitative evaluation of the thermal decomposition behavior and the stability of materials and provide reaction kinetics data over a broad temperature region [17,18]. However, the effect of the acetylation on the thermal decomposition kinetics of wood has not yet been assessed. The objective of this study was to compare and characterize slicewood that was VA-acetylated using potassium carbonate and potassium acetate catalysts by a vapor phase reaction. To the best of our knowledge, this is not only the first work addressing differences in VA-acetylated slicewood using different alkaline catalysts, but also the first work providing the thermal decomposition kinetics of acetylated slicewood by the model-free iso-conversional methods.

## 2. Materials & Methods

### 2.1. Materials

Japanese cedar (*Cryptomeria japonica* D. Don) sapwood (20–30 years old) was supplied by the experimental forest of the National Taiwan University. The dimensions of the slicewood samples were 3 mm (R) × 12 mm (T) × 58 mm (L). Oven-dried wood specimens that had straight longitudinal grain, were free of defects, and had modulus of elasticity (MOE) values from 5.5 to 7.0 GPa were selected for this study. All of the samples were used after extraction in a Soxhlet apparatus for 24 h with a 1:2 (*v*/*v*) mixture of ethanol and toluene followed by washing with distilled water. The extracted slicewood was dried at 105 °C for 12 h, and the oven-dried weights were measured. Vinyl acetate (VA), acetic anhydride (AA), dimethylformamide (DMF), potassium acetate (PA) (Sigma–Aldrich Chemical Co, St. Louis, MO, USA), potassium carbonate (PC) (Merck Chemical Co., Darmstadt, Germany), and other chemicals and solvents used in this experiment were of the highest quality available.

### 2.2. Acetylation

The slicewood was impregnated with different concentrations (0.05, 0.1, 0.25, and 0.5 M) of PC or PA solutions under reduced pressure for 30 min and then impregnated under atmospheric pressure for another 1 h. The impregnated samples were dried at 105 °C for 12 h. The catalyst content in the wood materials was calculated from the following equation:

Catalyst content (%) = [(w_1_ − w_0_)/w_0_] × 100 (1)

where w_0_ and w_1_ are the weight of an oven-dried sample before and after immersion, respectively.

The impregnated slicewood was acetylated with VA using a vapor phase reaction. The reaction used 20 mmol of VA and 20 mL of DMF per gram of oven-dried wood. All reactions were conducted at different temperatures (78 °C and 140 °C) for 0–24 h to obtain acetylated slicewood samples with different degrees of modification. At the end of the reaction, the acetylated samples were washed with distilled water and Soxhlet-extracted with acetone for 8 h. Finally, the acetylated samples were dried at 105 °C for 12 h, and the oven-drying method was used to calculate the weight percent gain (WPG) of the acetylated samples. In addition, for comparison purposes, a conventional acetylation process with AA by vapor phase reaction was also performed in this study, following the detailed procedure described in our previous study [10].

### 2.3. Flexural Properties

The modulus of rupture (MOR) and modulus of elasticity (MOE) of the specimens were determined by a three-point static bending test with a loading speed of 1.28 mm/min and a span of 48 mm (the specimen size was 3 mm × 12 mm × 58 mm) according to the American society for testing and materials (ASTM) standard D790 [19]. The samples were oven-dried at 105 °C for 12 h prior to testing. All tests were carried out in an air-conditioned room at 20 °C. Nine specimens were used for each determination.

### 2.4. Dynamic Mechanical Analysis

The dynamic viscoelastic properties of the slicewood samples were measured using a single-cantilever bending test (DMA 8000, PerkinElmer, Buckinghamshire, UK) at a heating rate of 5 °C/min and a frequency of 1 Hz. The storage modulus (*E*′) and loss modulus (*E*″) were recorded over a temperature range of −180 °C to 300 °C. The dimensions of the samples were 30 mm (L) × 12 mm (T) with a thickness of 3 mm.

### 2.5. Attenuated Total Reflectance Fourier Transform Infrared (ATR-FTIR) Spectral Measurements

ATR-FTIR spectra were recorded on a Spectrum 100 FTIR spectrometer (PerkinElmer, Buckinghamshire, UK) equipped with a deuterated triglycine sulfate (DTGS) detector and a MIRacle ATR accessory (Pike Technologies, Madison, WI, USA). The spectra were collected by co-adding 32 scans at a resolution of 4 cm^−1^ in the range of 650 to 4000 cm^−1^. 

### 2.6. Solid-State Cross-Polarization Magic Angle Spin (CP/MAS) Carbon-13 Nuclear Magnetic Resonance (^13^C-NMR) Analysis

The powder samples were examined by CP/MAS ^13^C-NMR. The spectra were recorded on a Bruker DSX-400WB FT-NMR spectrometer (Brucker, Bremen, Germany) with a sampling frequency of 100 MHz. The chemical shifts were calculated relative to tetramethylsilane (TMS).

### 2.7. Thermogravimetric Analysis (TGA)

A PerkinElmer Pyris 1 TG analyzer (Shelton, CT, USA) was used. Measurements of 3 mg samples were carried out in a nitrogen atmosphere (20 mL/min) from 50–600 °C. The heating rate was set to 5, 10, 20, 30, and 40 °C/min. The kinetic parameters were calculated based on the data obtained by the model-free iso-conversional methods. The conversion rate *α* is defined as:

*α* = (W_0_ − W*_t_*)/(W_0_ − W_∞_) (2)

where W_0_ is the initial weight of the sample, W_∞_ is the final residual weight, and W*_t_* is the weight of the oxidized or pyrolized sample at time *t*. The common iso-conversional methods used in this study include the methods of Friedman (Equation (3)), Flynn-Wall-Ozawa (F-W-O) (Equation (4)), the modified Coats-Redfern (C-R) (Equation (5)), and Starink (Equation (6)). The methods are represented by the following equations:

ln(d*α*/d*t*) = ln[*Af*(*α*)] − *E*_a_/(*RT*) (3)

log*β* = log[*AE*_a_/(*Rg*(*α*))] − 2.315 − 0.4567*E*_a_/(*RT*) (4)

ln{*β*/[*T*^2^(1 − 2*RT*/*E*_a_)]} = ln{–*AR*/[*E*_a_ln (1 − *α*)]} − *E*_a_/(*RT*) (5)

ln(*β*/*T*^1.8^) = *C*_s_ − 1.0037(*E*_a_/*RT*) (6)

where *α* is the conversion rate, *A* is the pre-exponential factor (min^−1^), *f* (*α*) is the reaction model, *E*_a_ is the apparent *E*_a_ (kJ/mol), *R* is the gas constant (8.314 J/K·mol^−1^), *T* is the absolute temperature (K), *β* is the heating rate, *g* (*α*) is a function of the conversion, and *C*_s_ is a constant [16,17,18]. Therefore, for a given conversion, linear relationships are observed by plotting ln(d*α*/d*t*), log*β*, ln(*β*/*T*^2^), and ln(*β*/*T*^1.8^) versus 1/*T* at different heating rates; the *E*_a_ is calculated from the slope of the straight line [16,17,18].

In addition to *E*_a_, the reaction order is also an important parameter for the thermal decomposition of wood [17]. The reaction order in this study was calculated based on the Avrami theory (Equation (7)):

ln[−ln(1 − *α*)] = ln*A* − *E*_a_/*RT* − *n*ln*β* (7)

where *n* represents the reaction order. For a given temperature, a linear relationship is observed by plotting ln[−ln(1 − *α*)] versus ln*β* at different temperature heating rates, and the reaction order is deduced from the slope of the line [17].

### 2.8. Statistical Analyses

All results were expressed as the means ± standard deviation (SD). The significance of the differences was calculated using Scheffe’s test; *p* values < 0.05 were considered to be significant.

## 3. Results and Discussion

### 3.1. Effects of the Reaction Temperature, Reaction Time, and Catalyst Content

**Figure 1:**
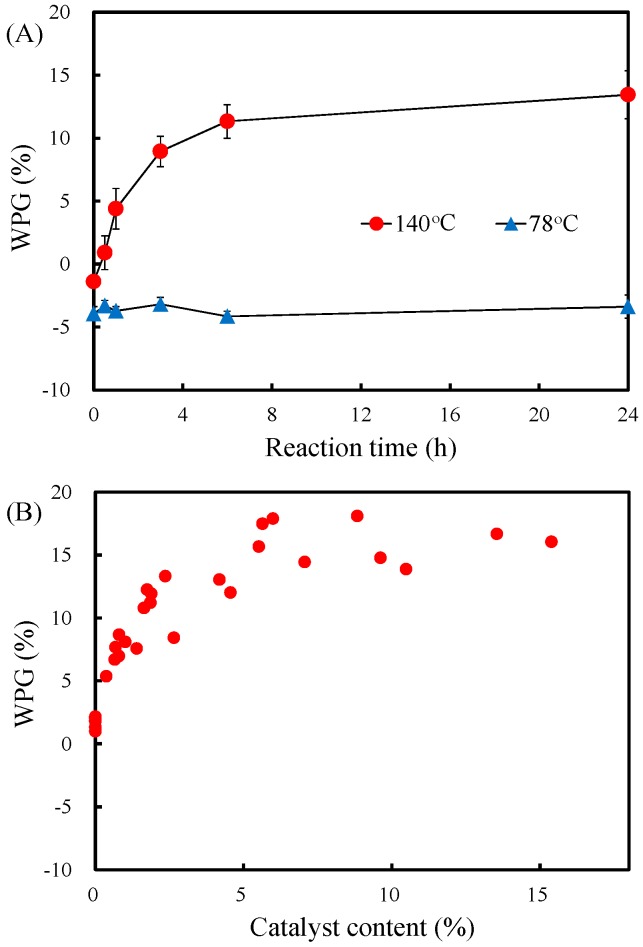
(**A**) The weight percent gain (WPG) of vinyl acetate (VA)-acetylated slicewood reacted at 78 °C and 140 °C as a function of reaction time. Values are mean ± standard deviation (SD) (*n* = 9); (**B**) Effect of potassium carbonate content on the WPG of VA-acetylated slicewood by the reaction at 140 °C for 6 h.

The effects of several reaction parameters including reaction temperature, reaction time, and catalyst content on the weight percent gain (WPG) of the slicewood acetylation with VA were investigated. Figure 1A shows the WPG of the slicewood that was acetylated with VA in 5 wt % PC at 78 and 140 °C for 0–24 h. The results clearly revealed that the change in WPG of the VA-acetylated slicewood at 78 °C was not significant. This phenomenon is likely caused by insufficient thermal energy for acetylation at the reaction temperature of 78 °C. When reaction temperature raised to 140 °C, expectedly, the WPG of VA-acetylated slicewood increases up to around 9.0% at the reaction time of 3 h. Once the reaction time reached 24 h, the WPG achieved 13.4%.

In addition, Figure 1B shows the WPG of the VA-acetylated slicewood with different PC contents at 140 °C for 6 h. The results revealed that the WPG of the VA-acetylated slicewood without catalyst was only 2%. As suggested by Çetin and Ozmen [11], Çetin et al. [20], and Jebrane and Sèbe [12], the VA-acetylated slicewood without catalyst gained hardly any weight. However, the WPG of VA-acetylated slicewood increases significantly to 5–9% when the PC content increased to 1%, and then the WPG increases with increasing PC contents up to 5%. This phenomenon can be explained that the addition of catalyst improves significantly the efficiency of the acetylation. Once the PC content exceeded 5%, there was no further increased in the WPG. Accordingly, the 5% of catalyst content was an optimum condition for the wood acetylation in this study.

### 3.2. WPG, MOE, and MOR of VA-Acetylated Slicewood

The WPG, modulus of elasticity (MOE), and modulus of rupture (MOR) of the VA-acetylated slicewood with two different catalysts, PC and PA, are shown in Table 1 as a function of the reaction time. The WPG of the VA-acetylated slicewood increased with the reaction time for both catalysts. At a reaction time of 0 h (i.e., the time point at which the reaction temperature reached 140 °C), the WPG of VA-acetylated slicewood with PC and PA was −1.4% and −3.5%, respectively. This phenomenon is likely caused by the degradation and leaching of wood components (hemicellulose and lignin) during immersion of the wood in the alkaline catalyst solutions, leading to a mass loss of wood. In addition, the WPG of VA-acetylated slicewood with PC (pH 11.6) increased to 11.3% at the reaction time of 6 h, and then level off. In contrast, the WPG of VA-acetylated slicewood with PA (pH 8.4) increased progressively with increasing the reaction time. However, the VA-acetylated slicewood with PC exhibited higher WPG than that with PA at all reaction time points. These results showed that PC as catalyst is more efficient than PA to acetylate wood with VA; similar result was observed by Çetin et al. [20].

Table 1 also shows that there were no significant differences in MOE and MOR values between unmodified and acetylated slicewood for both catalysts used. This result revealed that the bending properties of the VA-acetylated slicewood were not influenced by the degree of modification and the type of catalyst used in this study. As suggested by Birkinshaw and Hale [21] and Rowell and Banks [22], acetylation with AA did not significantly affect the mechanical properties of the softwoods (pine, lime, spruce, and larch).

### 3.3. Functional Groups and Calibrated WPG of VA-Acetylated Slicewood

**Figure 2:**
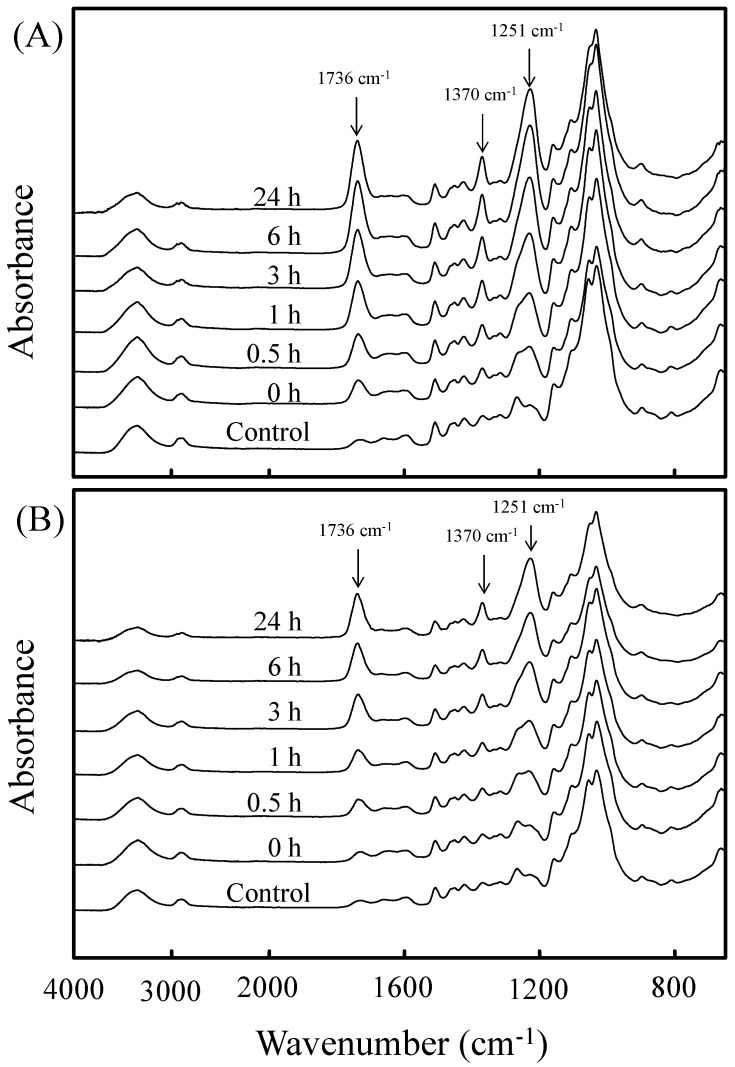
The attenuated total reflectance Fourier transform infrared (ATR-FTIR) spectra of VA-acetylated slicewood with potassium carbonate (PC) (**A**) and potassium acetate (PA) (**B**) as a catalyst at various reaction time.

Figure 2 shows the ATR-FTIR spectrum of VA-acetylated slicewood with two catalysts at various reaction time. Regardless of catalyst type, the increase in the absorption bands of acetyl group at 1736 cm^−1^ (-OCOCH_3_, C=O), 1370 cm^−1^ (-OCOCH_3_, C-H), and 1251 cm^−1^ (-OCOCH_3_, C-O) were observed after the VA acetylation. Additionally, the intensity of these absorption bands increased significantly with increasing reaction time. This result confirms that slicewood was successfully acetylated with vapor phase VA using PC or PA as catalyst. Fávaro et al. [23], Hung and Wu [24], and Khalil et al. [25] also reported a similar increasing trend of FTIR spectrum for AA-acetylated woody materials. Compared with different catalysts used, it can be seen that the signal intensity of the acetyl group of VA-acetylated slicewood with PC (Figure 2A) was higher than that with PA (Figure 2B). This phenomenon agreed with results of the WPG of the VA-acetylated slicewood, and again confirmed that the PC as catalyst is more efficient than PA to acetylate slicewood with VA.

**Figure 3:**
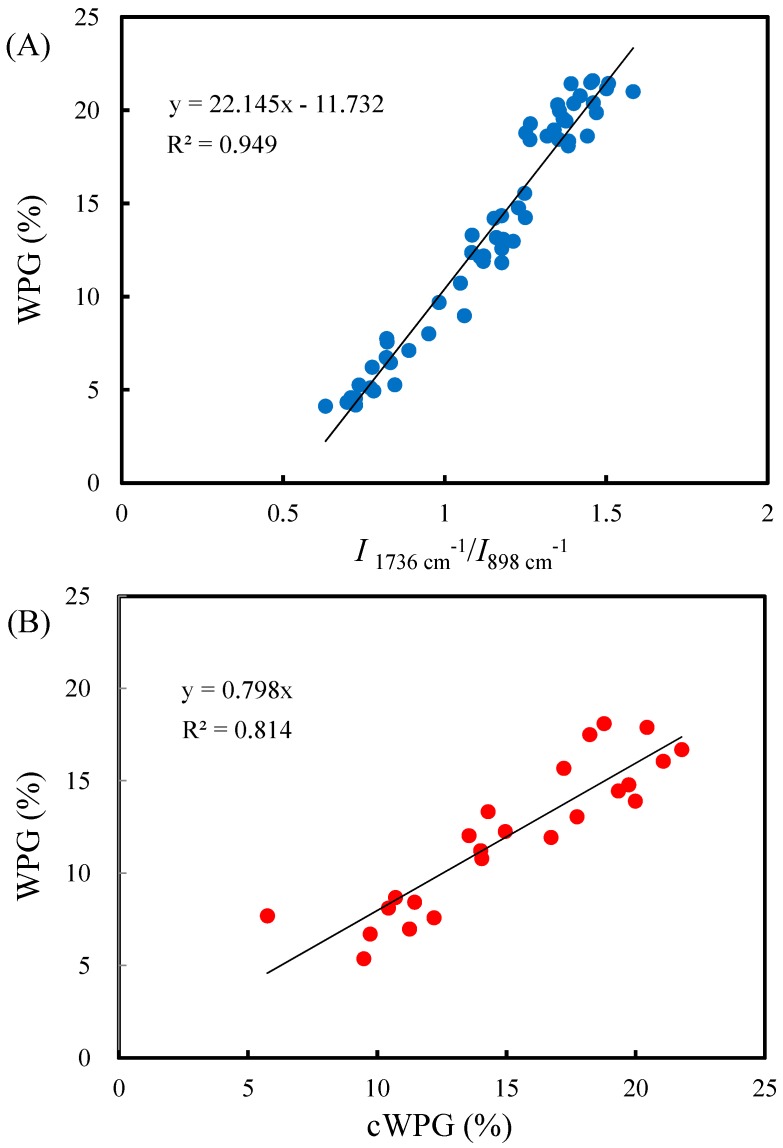
The correlations between the intensity of *I*_1736 cm__−1_/*I*_898 cm__−1_ and WPG of acetic anhydride (AA)-acetylated slicewood (**A**) and between the WPG and calibrated WPG (cWPG) of VA-acetylated slicewood (**B**) by vapor phase reaction for 6 h.

Furthermore, the WPG of VA-acetylated slicewood with PC or PA calculated by weight method may be underestimated because alkali-soluble extractives of wood components could be leaching during pretreatment with catalyst solutions. To overcome this problem, the relationship between the WPG of non-pretreatment AA-acetylated slicewood and their FTIR absorption intensities of acetyl ester group at 1736 cm^−1^ was further investigated to calibrate the WPG of VA-acetylated slicewood. Figure 3A shows the correlation between *I*_1736 cm_^−1^/*I*_898 cm_^−1^ and the WPG of the AA-acetylated slicewood. Linear regression analysis revealed a highly linear correlation (*R*^2^ = 0.949) between the two selected items. This result indicated that the WPG of acetylated wood could be accurately evaluated by the FTIR method. Therefore, the calibrated WPG (cWPG) of VA-acetylated slicewood was evaluated by *I*_1736 cm_^−1^/*I*_898 cm_^−1^ and compared to the WPG calculated by the weight method. As shown in Figure 3B, it was found that the slope of linear regression (*R*^2^ = 0.814) between WPG and cWPG of VA-acetylated slicewood was approximately 0.8. In other words, the WPG of VA-acetylated slicewood by the weight method was underestimated by 20%.

### 3.4. Reactive Characteristics of VA-Acetylated Slicewood

**Figure 4:**
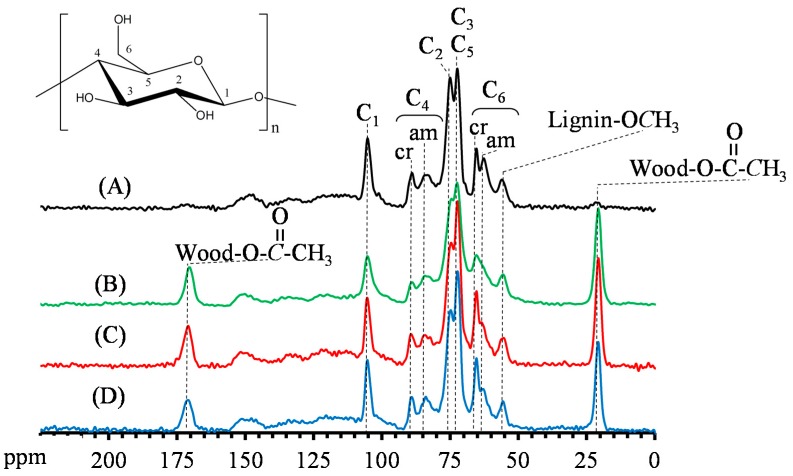
Solid state carbon-13 nuclear magnetic resonance (^13^C-NMR) spectra of unmodified slicewood (**A**), AA-acetylated slicewood (WPG 21%) (**B**), and VA-acetylated slicewood (cWPG 18%) with PC (**C**) and PA (**D**).

In this study, solid state CP/MAS ^13^C-NMR was used to elucidate the reactive characteristics of slicewood before and after VA acetylation. Figure 4 illustrates various characteristic carbohydrate patterns, including C1 (105.2 ppm), C4 (88.9 ppm and 83.3 ppm), C2 (75.0 ppm), C3, C5 (72.4 ppm), and C6 (65.3 ppm and 62.7 ppm) for the unmodified slicewood [7,10,24,26].

The signals of hemicellulose carbons were also found to resonate in the same field as that of the cellulose carbons, meaning that it could not be precisely identified among them [10]. The chemical shift at 56.1 ppm and the broad signal between 125 and 160 ppm corresponded to the methoxy groups and aromatic rings of lignin, respectively. In addition, the peaks at 170.9 and 20.7 ppm, which were assigned to the acetyl group, were also found in all the spectra from the AA- and VA-acetylated slicewood with all the catalyst. However, the intensity of the peaks at 75.0 (C2) and 62.7 (amorphous C6) ppm decreased and shifted downfield after acetylation, whereas there was no significant change observed at 72.4 ppm (C3). Compared with the AA-acetylated slicewood, the solid state ^13^C-NMR spectra of VA-acetylated slicewood exhibited a remarkable decrease in the signal intensity of the C2 position (75.0 ppm) for the VA-acetylated slicewood. In contrast, the signal intensity of amorphous C6 (62.7 ppm) of VA-acetylated slicewood was higher than that of AA-acetylated one. These results indicated that the order of the reactivity of the cellulose hydroxyl groups was C2–OH > C6–OH > C3–OH when the slicewood was modified with VA, while in the case of AA acetylation, the order of the reactivity was C6–OH > C2–OH > C3–OH. Similar results were also reported by Jebrane and Sèbe [12]. In addition, there were no significantly differences between the ^13^C-NMR spectra of VA-acetylated slicewood with PC and PA. This result implied that the accessibility of the modifying agent for VA acetylation was independent of the types of catalysts used in this study.

### 3.5. Viscoelastic Properties of Acetylated Slicewood

**Figure 5:**
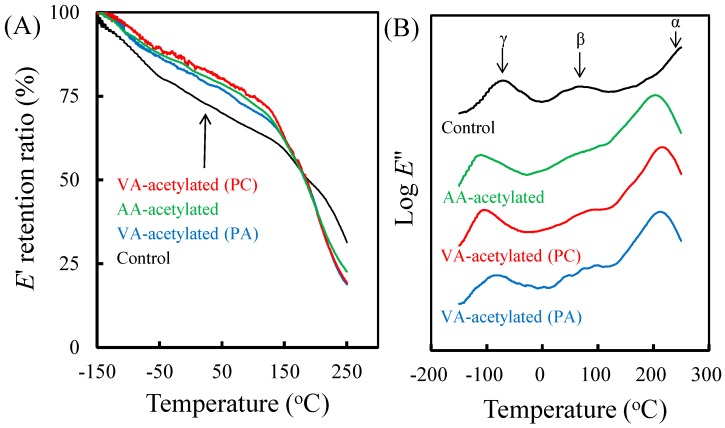
The storage modulus (*E*′) retention ratio (**A**) and loss modulus (*E*″) (**B**) of unmodified slicewood, AA-acetylated slicewood (WPG 21%), and VA-acetylated slicewood (cWPG 18%) with PC and PA.

To understand the difference in viscoelastic properties of slicewood after acetylation, the dynamic mechanical analysis (DMA) was carried out in this study. As shown in Figure 5A, the *E*′ retention ratio of acetylated slicewood was significantly different from the unmodified slicewood. The *E*′ retention ratio of AA- and VA-acetylated slicewood was higher than that of unmodified slicewood (control) at temperature below 150 °C. However, all the acetylated slicewood exhibited a dramatic decrease in the *E*′ retention ratio as the temperature increased beyond 150 °C. Obataya et al. suggest that some of hydrogen bonds between the amorphous molecules are severed as the acetyl groups (an internal plasticizer) are introduced into the system, resulting in an increase in the mobility of the amorphous molecules at elevated temperature [27].

On the other hand, Figure 5B reveals that the *E″* of the unmodified slicewood shows three distinct relaxation processes labeled *α*, *β*, and *γ* in order of decreasing temperature, and which they are assigned to the micro-Brownian motions of lignocellulose polymers in the non-crystalline regions, the motions of lignin and/or lignin-hemicellulose complexes plasticized with water, and the motions of the methylol groups of the lignocellulose polymers in the amorphous zones, respectively [6,27,28,29]. It was observed that both *α* and *γ* transitions of all the acetylated slicewood exhibited the same tendency, i.e., shifting to lower temperature. Again, this result indicated that the lignocellulose polymers mobility was improved in the non-crystalline regions because some of the hydrogen bonds between the amorphous molecules were severed with the introduction of the acetyl groups. In addition, comparison with the unmodified slicewood, the *β* transition of all the acetylated slicewood was remarkably vanished, which revealed a reduction in the hygroscopicity of the slicewood after acetylation. Meanwhile, a new *β* transition appeared at around 70 °C for the acetylated slicewood, especially treatment with VA. A similar result was also reported by Jebrane et al. [7], who suggested that cellulose sites in wood were more attacked by VA than by AA. However, there are no significant differences in the viscoelastic properties between PC- and PA-catalyzed acetylations of slicewood.

### 3.6. Thermal Decomposition Kinetics of Acetylated Slicewood

**Figure 6:**
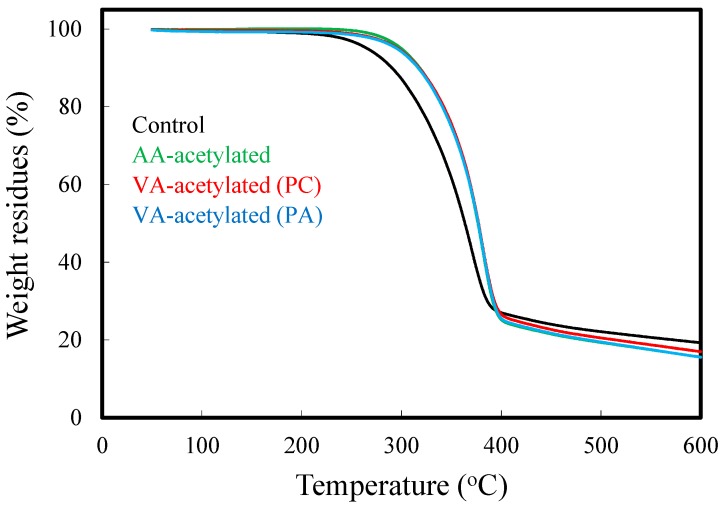
Thermogravimetric curves of unmodified slicewood, AA-acetylated slicewood (WPG 18%), and VA-acetylated slicewood (cWPG 18%) with PC and PA.

Figure 6 shows the TGA curves of unmodified slicewood (control), AA-acetylated slicewood, and VA-acetylated slicewood with two different catalysts. The TGA curve of unmodified slicewood exhibited a gradually increasing weight loss above 200 °C and a maximum weight loss was obtained around 350 °C. In contrast, the pyrolysis of the AA- and VA-acetylated slicewood was delayed ca. 10 °C, compared to the unmodified slicewood, indicating that the thermal stability of slicewood could be effectively enhanced through acetylation. A similar result was obtained by Xu et al. [13], Özmen et al. [14], and Wei et al. [15]. Additionally, the solid residue of all acetylated slicewood (15%–17%) was lower than the unmodified slicewood (19.3%) at 600 °C, and it is possible that the acyl groups in the acetylated wood were eliminated with the volatile products and did not convert to the char [5].

**Figure 7:**
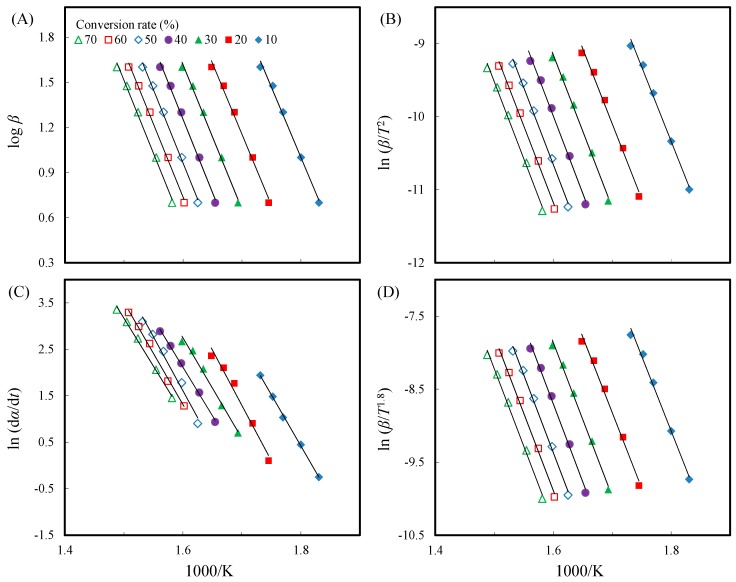
Typical iso-conversional plots of Flynn-Wall-Ozawa (F-W-O) (**A**); modified Coats-Redfern (C-R) (**B**); Friedman (**C**); and Starink (**D**) methods for unmodified slicewood.

To further understand the thermal decomposition kinetics of unmodified and acetylated slicewood, the model-free iso-conversional methods were used. The plots of the iso-conversional Friedman, Flynn-Wall-Ozawa (F-W-O), modified Coats-Redfern (C-R), and Starink methods describe a general trend in the *E*_a_. As an example, typical plots based on the models F-W-O, modified C-R, Friedman, and Starink for unmodified slicewood are presented in Figure 7. The slopes of the fitted lines between 10% and 70% conversion were nearly parallel, indicating the approximate *E*_a_ at different conversion methods. The acetylated slicewood plots are similar to those presented in Figure 7 (not shown). As reported by Yao et al., a single possible reaction mechanism takes place between 10% and 70% conversion rates in natural fibers, which might provide a simplified and more meaningful method to explain the thermal decomposition behavior of natural fibers [16]. Hence, in this study, the apparent *E*_a_ was estimated in this conversion range. The correlation coefficient (*R*^2^) and the *E*_a_ values calculated from the above-mentioned methods are listed in Table 2.

As shown in Table 2, the *E*_a_ values of unmodified slicewood calculated by F-W-O method are between 170 and 178 kJ/mol (at the conversion range of 10–70%), and the *E*_a_ value increases with increasing conversion rate up to 40%. As found by Yao et al., the average apparent *E*_a_ of natural fibers calculated between 10% and 60% by the F-W-O method was 171.5 kJ/mol [16]. In the present study, the average apparent *E*_a_ of unmodified slicewood is 175 kJ/mol, which is in good agreement with the literature data. Moreover, the average apparent *E*_a_ of the acetylated slicewood was 196, 195, and 184 kJ/mol for AA- (WPG 18%), VA-acetylated slicewood (cWPG 18%) with PC and PA, respectively. The *E*_a_ is defined as the minimum energy that must be overcome to start a chemical reaction [30]. As the *E*_a_ values increased, the minimum energy required to start the thermal decomposition was also elevated. Similar results from modified C-R, Friedman, and Starink methods also confirm this observation (Table 2). The average *E*_a_ values calculated in the conversion range of 10–70% were 174–183, 195–208, 194–200, and 183–186 kJ/mol for unmodified, AA-acetylated, and VA-acetylated slicewood with PC and PA, respectively. A statement of Brown et al. and Yao et al. should be remembered, according to which different kinetic analysis methods are complementary, rather than competitive [16,31]. Thus, a suitable apparent *E*_a_ range could be obtained by combining all observations in Table 2. Compared with different catalysts used, it can be seen that the average apparent *E*_a_ of VA-acetylated slicewood with PC was higher than that with PA. This result indicated that the VA-acetylated slicewood with PC exhibited more thermal stability than that with PA.

**Figure 8:**
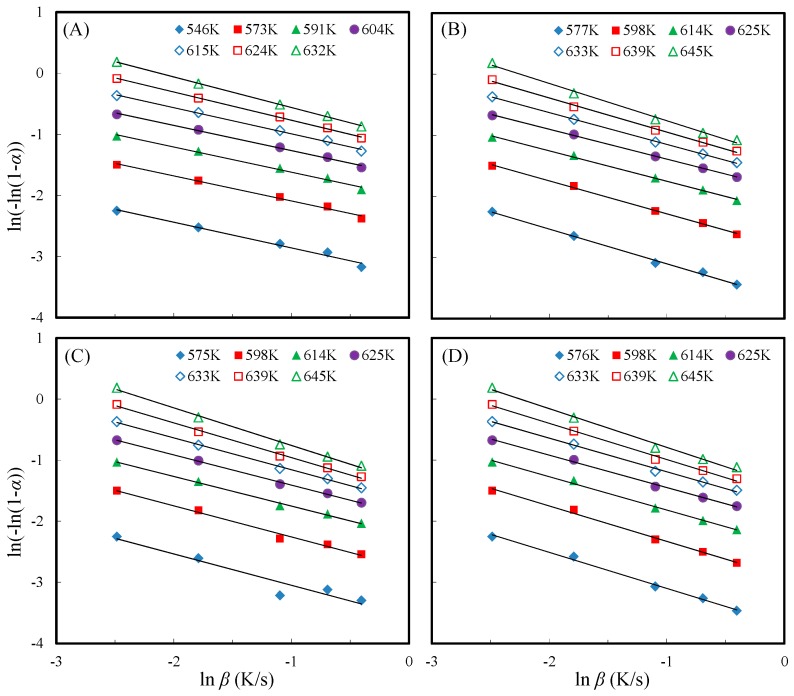
Regression lines to reaction order proposed by Avrami theory for unmodified slicewood (**A**); AA-acetylated slicewood (WPG 18%) (**B**); and VA-acetylated slicewood (cWPG 18%) with PC (**C**) and PA (**D**).

Moreover, to estimate the dependence of the reaction order (*n*) on the decomposition temperature during the major thermal decomposition process, seven decomposition temperatures were employed at five heating rates (5, 10, 20, 30, and 40 K/min). The seven decomposition temperatures were the temperature of conversion between 10% and 70% at a heating rate of 5 K/min. Based on the Avrami theory, the regression lines of the unmodified and all acetylated slicewood are illustrated in Figure 8. The calculated reaction order and corresponding *R*^2^ values are also presented in Table 2. It is remarkable that most *R*^2^ values are higher than 0.99. Thus, the Avrami theory is suitable to evaluate the reaction order of unmodified and acetylated slicewood. The reaction order was range of 0.41–0.50, 0.49–0.61, 0.48–0.65, and 0.53–0.63 for unmodified, AA-acetylated, and VA-acetylated slicewood with PC and PA, respectively (Table 2). It has been reported that the reaction orders of wood waste, corn straw, and rice husks are 0.420, 0.365, and 0.539, respectively [17,32]. The reaction order of unmodified slicewood was found in a similar range as the literature. In addition, the reaction order of slicewood increased after AA or VA acetylation, indicating that introduction of acetyl groups into the wood may have changed the thermal decomposition path. Accordingly, the acetylation improves the thermal stability of slicewood, and the thermal stability of VA-acetylated slicewood with PC was better than that with PA.

## 4. Conclusions

Alkali-catalyzed acetylation with VA by vapor phase reaction could be used as an alternative process for wood acetylation, and in which the optimal catalyst loading was 5%. There are no significant differences in bending and viscoelastic properties between PA- and PC-catalyzed acetylations of slicewood. The reactivity of the hydroxyl groups in the cellulose was sensitive at the C2 reactive site for the alkali-catalyzed acetylation of slicewood with VA, while the C6 reactive site was sensitive for the conventional acetylation with AA. The TG curves of the acetylated slicewood shifted to a higher temperature field compared to the unmodified slicewood. The average apparent *E*_a_ of the acetylated slicewood was higher than that of the unmodified slicewood, and the VA-acetylated slicewood with PC has higher apparent *E*_a_ than that with PA. For the range of conversion rates (10–70%) investigated, the reaction order was 0.41–0.50, 0.49–0.61, 0.48–0.65, and 0.53–0.63 for unmodified, AA-acetylated, and VA-acetylated slicewood with PC and PA, respectively. These results indicate that the thermal stability of slicewood could be enhanced by vapor phase VA acetylation with an alkaline catalyst.

## Figures and Tables

**Table 1:** The weight percent gain (WPG), modulus of elasticity (MOE), and modulus of rupture (MOR) of vinyl acetate (VA)-acetylated slicewood with two different catalysts by the reaction at 140 °C for 0–24 h.

Reaction Time (h)	Potassium Carbonate (5%)			Potassium Acetate (5%)		
	WPG (%)	MOE (GPa)	MOR (MPa)	WPG (%)	MOE (GPa)	MOR (MPa)
Control	−	6.5 ± 0.4 ^a^	79 ± 14 ^a^	−	6.5 ± 0.4 ^a^	79 ± 14 ^a^
0	−1.4 ± 0.4 ^e^	6.6 ± 1.3 ^a^	91 ± 18 ^a^	−3.5 ± 1.1 ^e^	6.9 ± 1.0 ^a^	100 ± 20 ^a^
0.5	0.9 ± 1.3 ^d^	5.8 ± 0.6 ^a^	80 ± 2 ^a^	−0.7 ± 0.8 ^d^	6.1 ± 1.1 ^a^	78 ± 18 ^a^
1	4.4 ± 1.6 ^c^	5.8 ± 0.9 ^a^	80 ± 14 ^a^	0.2 ± 0.8 ^d^	6.5 ± 0.9 ^a^	105 ± 17 ^a^
3	9.0 ± 1.2 ^b^	5.6 ± 0.6 ^a^	94 ± 33 ^a^	3.6 ± 1.3 ^c^	5.1 ± 2.5 ^a^	70 ± 33 ^a^
6	11.3 ± 1.2 ^a^	6.7 ± 1.2 ^a^	89 ± 24 ^a^	9.4 ± 1.1 ^b^	5.1 ± 1.3 ^a^	78 ± 13 ^a^
24	13.4 ± 1.9 ^a^	5.5 ± 0.8 ^a^	76 ± 20 ^a^	12.9 ± 1.1 ^a^	6.6 ± 1.4 ^a^	88 ± 23 ^a^

Values are the means ± standard deviation (SD) (*n* = 9). Different superscript letters within a column indicate significant differences at *p* < 0.05.

**Table 2:** Apparent activation energy and reaction order of unmodified slicewood, acetic anhydride (AA)-acetylated slicewood (WPG 18%), and vinyl acetate (VA)-acetylated slicewood (cWPG 18%) with potassium carbonate (PC) and potassium acetate (PA) calculated by the methods of Flynn-Wall-Ozawa (F-W-O), modified Coats-Redfern (C-R), Friedman, Starink, and Avrami.

Slicewood	Methods	Units	Conversion Rates							
			10%	20%	30%	40%	50%	60%	70%	Mean
Unmodified	F-W-O	*E*_a_ (kJ/mol)	170	172	175	178	177	176	177	175
(Control)		*R*^2^	0.994	0.992	0.995	0.995	0.996	0.996	0.996	–
	Modified C-R	*E*_a_ (kJ/mol)	169	171	174	176	176	175	175	174
		*R*^2^	0.993	0.991	0.995	0.995	0.996	0.996	0.995	–
	Friedman	*E*_a_ (kJ/mol)	183	198	180	174	193	183	172	183
		*R*^2^	0.999	0.978	0.989	0.998	0.984	0.996	0.996	–
	Starink	*E*_a_ (kJ/mol)	170	172	175	177	176	175	176	174
		*R*^2^	0.993	0.991	0.995	0.995	0.996	0.996	0.996	–
	Avrami	Reac. order	0.42	0.41	0.41	0.41	0.43	0.46	0.50	0.43
		*R*^2^	0.984	0.992	0.992	0.996	0.997	0.999	≈1	–
AA-acetylated	F-W-O	*E*_a_ (kJ/mol)	182	191	198	202	201	199	199	196
		*R*^2^	0.999	≈1	0.999	0.999	0.999	≈1	≈1	–
	Modified C-R	*E*_a_ (kJ/mol)	181	190	197	202	200	198	199	195
		*R*^2^	0.999	≈1	0.999	0.999	0.999	≈1	0.999	–
	Friedman	*E*_a_ (kJ/mol)	201	232	205	229	191	192	206	208
		*R*^2^	0.980	0.980	0.997	0.992	0.998	0.999	0.997	–
	Starink	*E*_a_ (kJ/mol)	182	191	198	202	201	198	199	196
		*R*^2^	0.999	≈1	0.999	0.999	0.999	≈1	0.999	–
	Avrami	Reac. order	0.57	0.54	0.50	0.49	0.52	0.56	0.61	0.54
		*R*^2^	0.997	0.998	0.998	0.999	≈1	0.997	0.994	–
VA-acetylated	F-W-O	*E*_a_ (kJ/mol)	175	186	194	201	202	201	203	195
(PC)		*R*^2^	0.988	0.998	0.999	≈1	≈1	≈1	≈1	–
	Modified C-R	*E*_a_ (kJ/mol)	174	186	194	201	202	201	203	194
		*R*^2^	0.987	0.997	0.999	0.999	≈1	≈1	≈1	–
	Friedman	*E*_a_ (kJ/mol)	172	206	202	196	205	206	213	200
		*R*^2^	0.980	0.995	0.996	0.999	0.999	0.996	0.999	–
	Starink	*E*_a_ (kJ/mol)	175	186	194	201	202	201	203	195
		*R*^2^	0.987	0.997	0.999	0.999	≈1	≈1	≈1	–
	Avrami	Reac. order	0.51	0.51	0.48	0.52	0.57	0.62	0.65	0.55
		*R*^2^	0.926	0.989	0.996	0.998	0.998	0.997	0.996	–
VA-acetylated	F-W-O	*E*_a_ (kJ/mol)	163	173	183	189	194	193	194	184
(PA)		*R*^2^	0.995	0.995	0.995	0.995	0.996	0.996	0.994	–
	Modified C-R	*E*_a_ (kJ/mol)	161	172	182	189	194	193	193	183
		*R*^2^	0.995	0.994	0.995	0.995	0.995	0.995	0.993	–
	Friedman	*E*_a_ (kJ/mol)	157	170	200	193	195	186	201	186
		*R*^2^	0.987	0.989	0.997	0.995	0.995	0.991	0.991	–
	Starink	*E*_a_ (kJ/mol)	162	172	182	189	194	193	193	184
		*R*^2^	0.995	0.994	0.995	0.995	0.995	0.995	0.994	–
	Avrami	Reac. order	0.59	0.58	0.55	0.53	0.55	0.59	0.63	0.57
		*R*^2^	0.995	0.995	0.996	0.996	0.996	0.995	0.992	–
